# Differences in Spatial Cognition and Motor–Cognitive Integration by Side of Onset in People with Parkinson’s Disease

**DOI:** 10.3390/brainsci16060619

**Published:** 2026-06-10

**Authors:** Ejew Beyla Kim, Morgan Brianna Patrick, Liang Ni, J. Lucas McKay, Madeleine Eve Hackney

**Affiliations:** 1 Department of Medicine, Division of Geriatrics and Gerontology, School of Medicine, Emory University, Atlanta, GA 30322, USA; ejewkim@gmail.com (E.B.K.); morgan.patrick1@vcuhealth.org (M.B.P.); liangni12@gmail.com (L.N.); 2 Department of Biomedical Engineering, Emory University and the Georgia Institute of Technology, Atlanta, GA 30332, USA; j.lucas.mckay@emory.edu; 3 Department of Biomedical Informatics, School of Medicine, Emory University, Atlanta, GA 30322, USA; 4 Department of Rehabilitation Medicine, School of Medicine, Emory University, Atlanta, GA 30322, USA; 5 Atlanta VA Center for Visual & Neurocognitive Rehabilitation, Atlanta, GA 30033, USA; 6 Birmingham/Atlanta VA Geriatric Research Education and Clinical Center, Atlanta, GA 30319, USA

**Keywords:** Parkinson’s disease, side of onset, spatial cognition, aging, unilateral

## Abstract

**Highlights:**

**What are the main findings?**

• LOPD tends to perform worse in single-task, dual-task, and/or dual-task cost TUG tasks within people with bilateral symptoms and postural instability, moderate to severe dementia, and more frequent freezing of gait.
• No significant differences were found in other spatial–cognitive, cognitive, or motor tasks, nor between unstratified onset groups.

**What are the implications of the main findings?**

• The level of impairment in PD may be a factor in revealing the side of onset differences in the TUG task.
• The effect of the side of PD onset may be detectable only by specific motor–cognitive tasks that utilize spatial cognition, rather than direct and traditional visuospatial tasks.

**Abstract:**

**Background:** Spatial cognition, a skill paramount to survival, is impaired in Parkinson’s disease (PD) but has been little researched. Spatial cognition is utilized during motor–cognitive integration, which impacts daily functioning and quality of life in PD. As PD is a unilateral-onset condition, spatial–cognitive and motor–cognitive ability may differ by side of onset. Spatial cognition is suggested to be modulated by the right hemisphere; thus, we hypothesize to observe worse spatial and motor–cognitive performance by people with left-onset PD (LOPD) than right-onset PD (ROPD). **Methods:** 216 participants with PD were recruited (LOPD = 107; M = 62; mean age = 69.80 ± 8.5). Spatial outcomes were collected via the body position spatial task (BPST), Reverse Corsi Blocks, and visuospatial items of the Montreal Cognitive Assessment (MoCA); motor–cognitive outcomes were collected by a Trails test, a Four Square Step Test (FSST), and a Timed Up and Go test. An independent *t*-test and the Mann–Whitney U test compared outcome variables between onset groups. **Results:** No significant differences were found between onset groups. Exploratory subgroup analyses revealed differences. Significantly worse performance by LOPD in single- and dual-task TUG was found within people with bilateral symptoms and postural instability (Hoehn & Yahr stage, >2; LOPD, N = 33; single, *p* = 0.001; dual, *p* = 0.021) and worse performance in single-task TUG in people with MoCA < 18 (LOPD, N = 5; single, *p* = 0.036) and people with freezing of gait (FOGQ, >0; LOPD, N = 14, *p* = 0.048). Significantly larger DTC by LOPD was found within frequent freezers (FOGQ, >3; LOPD, N = 9; *p* = 0.003). **Conclusions:** LOPD may tend to perform worse in motor–cognitive tasks among subgroups of those with more severe symptoms, i.e., those at later stages of disease. These findings may have implications for prognoses of those with LOPD versus ROPD and suggest that those with LOPD may have worse long-term outcomes in spatial cognition and motor–cognitive integration.

## 1. Introduction

Parkinson’s Disease (PD) is a neurodegenerative motor disorder caused by a loss of dopamine-inducing cells in the basal ganglia, resulting in progressive deterioration of motor and nonmotor functions. PD onset often occurs after 50 years of age and is becoming increasingly prevalent, with a projection of 25.2 million affected adults by 2050 [1]. The four cardinal signs of PD are bradykinesia, resting tremor, rigidity, and postural instability. Diagnosis is made when three out of four cardinal symptoms, along with clear benefits from antiparkinsonian medications, are present [2]. However, PD also involves a wide spectrum of nonmotor symptoms, such as cognitive and sleep issues. Management of cognitive symptoms is especially crucial. Many people with PD eventually experience cognitive decline, as 40% of PD have mild cognitive impairment (MCI) in the early stages of PD and 80% of PD end up with dementia at a later stage [3]. These cognitive symptoms negatively affect health. PD with cognitive impairment is associated with lower quality of life, caregiver burden [4], and increased comorbidities and hospital visits [5]. However, not much is known about the underlying mechanism of PD cognitive decline.

### 1.1. Spatial–Cognitive Decline in PD

Spatial cognition, which humans use to establish orientation in the surrounding environment and is paramount to survival, is often impaired in people with PD [6,7,8]. While spatial cognition mainly involves activity in the entorhinal cortex and hippocampus [9], studies found reduced brain activity in hippocampal regions and smaller entorhinal cortex volume in people with PD than healthy older adults of similar age [10,11]. Nonetheless, the mechanism underlying spatial–cognitive decline in PD is still under-researched.

### 1.2. Motor–Cognitive Integration and Spatial Cognition

Cognition, including spatial cognition, is not limited to purely cognitive activity but extends to motor activity. The literature increasingly presents overlapping activity of cognitive function during motor activity in PD [12], which is referred to as motor–cognitive integration [6]. Freezing of gait, a motor symptom that is a major cause of falls in PD and negatively impacts quality of life, has been found to be related to cognitive and executive function [13]. Rehabilitative strategies that train motor–cognitive integration were found to be beneficial for people with PD [14,15]. Effective dual-tasking, which is performing a mental task while also performing a motor task, necessitates the incorporation of sufficiently functional motor–cognitive integration. In PD, worse gait asymmetry [16], postural instability [17], gait speed, and gait variability [18] were found with the addition of a simultaneous cognitive task, and gait variability was further associated with executive function during dual-tasking [18], suggesting integrative work by motor and cognitive systems. Further, motor–cognitive dual-task training reduced dual-task interference and improved motor automation for PD, indicating quicker and smoother motor–cognitive integration [19].

Spatial cognition also extends to and is relevant for motor function. In PD, visuospatial measures were correlated with gait speed and stride and step lengths [20] and with deficits in daily living and motor functioning [21], including freezing of gait [22] and veering in gait that cause unstable walking [23]. Decline in visuospatial–cognitive scores over time was correlated with the decline in motor ability and worse severity of Postural Instability Gait Disorder in people with PD [24]. Performance on visuospatial processing tasks, e.g., Block Design and Matrix Reasoning, was associated with freezing [25]. Higher visuospatial–cognitive scores were correlated with lower FOG risk [26].

Importantly, deficits in spatial ability further affect the ability to perform tasks that demand motor–cognitive integration. Spatial cognition has been shown to be correlated with dual-tasking performance, for instance. Visuospatial ability was predictive of gait variability during walking in the preferred pace and dual-task condition [27]. Performance in spatial–cognitive tasks was correlated with impaired dual-task cost (DTC) values for motor–cognitive dual-task tests in people with PD [28,29]. In a study where older adults followed two different directional cues simultaneously—one using their body to sway left, right, front, or back; another clicking through an electronic grid to move a square either left, right, front, or back, or left, right, up, or down—greater postural instability was found when participants had to move the square left, right, up, or down, a different spatial orientation compared to the posture task and thus a spatial challenge. This showed that intact spatial ability was needed for successful postural–cognitive dual-tasking [30]. Overall, examining motor–cognitive function could reveal insights about spatial–cognitive function in PD.

### 1.3. Unilateral Onset in PD

A defining feature of PD is unilateral symptom onset, in which motor symptoms initially appear on one side of the body and then progress to bilateral presentation as the disease advances [31]. In the Hoehn and Yahr scale—a scale universally used to determine PD severity—unilateral symptoms are a defining feature for scores 1 and 1.5, while scores 2 and above require bilateral symptoms [32]. PD shows asymmetric patterns in functional activity via MRI and dopaminergic binding in striatal regions [33,34].

Some research identified clinical differences between left-onset PD (LOPD) and right-onset PD (ROPD). ROPD is associated with worse motor symptoms, daily life function, and complications from medication [35]. LOPD is associated with longer duration of illness, higher anxiety, and less magical ideation [36,37,38]. LOPD also presented worse cognitive symptoms, including executive function via the digital backward span task and baseline attention, but also greater improvement after sustained attentional training [38,39]. Yet, a recent meta-analysis generally compared LOPD and ROPD on symptom severity measures (UPDRS, MD-UPDRS, Hoehn and Yahr) and found a small effect size (g = 0.04, 95% CI = −0.05 to 0.12, *p* = 0.41), suggesting no significant effect of side of PD onset [40].

### 1.4. Spatial Cognition and Motor–Cognitive Integration by Side of PD Onset

Another review recently suggested that while many existing studies have utilized global cognitive scales to evaluate cognitive differences between LOPD and ROPD, a more precise focus on specific cognitive aspects may be needed to detect the cognitive deficits coming from asymmetrical symptomology [41]. In addition, the researchers presented that studies consistently indicate worse performance by LOPD than ROPD in the visuospatial domain [41]. However, this analysis was limited to the screening keywords asymmetry and lateralization and did not include other similar terms, such as side of onset. These other studies using the term “side of onset” introduce inconsistent results. One study found lower scores in the clock drawing test by LOPD without dementia when scored with the Rouleau method [42]; however, another study involving people with an H&Y less than 2.5 found no significant difference in the clock drawing test [43]. LOPD demonstrated worse performance in the Rey–Osterrieth complex figure test, which tests visuoconstructional ability, but this result was not consistent for people with a PD duration of 5 years and less [44]. No effect of the side of onset was found for performance in the Stick Construction task, another visuoconstructional assessment, in people with PD and H&Y stage less than 2 [45]. While these discrepancies suggest that an effect of side of onset may depend on PD characteristics, no study has specifically examined the effect of side of onset on spatial cognition based on different characteristic pools.

Moreover, most existing studies involve spatial tasks that are sedentary or limited to simple upper-limb movement. However, deficits in postural and lower-body movement have been associated with worse motor–cognitive integration [6,17] and are key to gait and balance issues that can lead to severe falls and reduced quality of life [46,47]; thus, the integration between motor function and spatial cognition, by evaluating spatial–cognitive function involving lower-body movement, may be important.

Studies on functional specialization, i.e., the concept that different brain regions specialize in certain functions, suggest that spatial cognition is modulated by the right hemisphere [48]. Though the extent of right-hemispheric specialization is unknown, cortical regions in the right hemisphere were found to be significantly more activated during spatial attention and target detection tasks in healthy adults [49]. Greater right-hemispheric activity was also found during a motor–cognitive dual-task of completing a cognitive test that involved spatial processing while walking [50].

### 1.5. Objective

Therefore, in our study, we present two branches of investigation: the impact of the side of PD onset that affects (a) spatial cognition and (b) the ability to perform motor–cognitive tasks that demand functional spatial cognition. For further exploratory analysis, we compared spatial–cognitive and motor–cognitive function between individuals with left versus right side of onset within the following groups: those with unilateral symptoms, bilateral symptoms, and bilateral symptoms and postural instability based on Hoehn and Yahr score; those with MoCA scores suggesting normal cognition, mild cognitive impairment, and moderate to severe cognitive impairment; and non-freezers, freezers, and frequent freezers. Stratifying using these three characteristics that describe the level of impairment in PD allows better isolation of the side of onset as the independent measure, as well as the opportunity to detect differences that may only occur at certain stages of the disease. Understanding the effect of the side of onset on spatial cognition and motor–cognitive integration could provide direction in learning the underlying mechanism of cognitive decline in PD.

### 1.6. Hypothesis

Therefore, using aggregated data from four clinical trials conducted in the Southeastern United States, we tested the hypothesis that people with LOPD, who are likely affected in their right hemispheric basal ganglia, including the posterior putamen and substantia nigra pars compacta [51,52], perform worse in both spatial–cognitive and motor–cognitive tasks than people with ROPD.

## 2. Materials and Methods

This study is a secondary data analysis using aggregated data from four different clinical trials that involved people with PD who participated in observational studies conducted from 2020 to today. The Emory University institutional review board (IRB) reviewed and approved the protocols of each study. In all studies, all participants provided informed written consent before participating.

Both veteran and non-veteran participants are included in this study. Veteran and non-veteran participants were recruited via foundation and senior facility outreach events, physician referrals, and word of mouth. Interested individuals were contacted via phone for screening and scheduling for the initial in-person assessment.

This study used data collected from 216 adults older than 50 years with PD from diverse racial groups and from greater metro Atlanta. Data collected was from a one-time study visit for baseline assessment. Questionnaires were completed by the participants themselves. Spatial–cognitive and motor–cognitive assessments were administered by trained research staff to measure mobility and cognition. In all studies, the PI, who is Movement Disorders Society Unified Parkinson Disease Rating Scale (MDS-UPDRS) [53] trained and certified, conducted clinical examinations to determine the side of onset and H&Y stage of PD of participants based on physical and motor functioning.

The spatial–cognitive outcomes included are the following: The Reverse Corsi Blocks test [54], the visuospatial section, the clock drawing test and the cube drawing test of the Montreal Cognitive Assessment [55], and the body position spatial task [6] (see Appendix A.1 for task description). Outcomes for motor cognitive integration were dual-task cost (DTC) of the Timed Up and Go (TUG) test [56], time to complete the cognitive version of TUG (TUG-Cognitive) [57], Four Square Step Test (FSST) [58], versions A and B of the trails test (Trail A and Trail B), and the difference in time to complete the two versions (Trails B-A) [59] (see Appendix A.2 for task description). While TUG-Cognitive is a measure of the time taken to perform the motor–cognitive dual task, TUG-DTC indicates how much more time was taken to perform the dual task compared to the single task. Other outcomes were the basic version of TUG (TUG-Simple) [60] and MoCA total score [55]. TUG-Simple measures the time taken to perform just the motor task. These other outcomes were included to provide further insight into the possible side of onset differences in solely motor ability and cognition, respectively.

### 2.1. Statistical Analysis

Independent *t*-tests were used to compare continuous variables of demographic and clinical characteristics and spatial–cognitive, motor–cognitive, motor, and cognitive outcomes for the entire sample. The chi-square test was used for categorical variables of demographic characteristics. Normality was examined using QQ distribution plots and the Shapiro–Wilk test. *p*-values were adjusted using the Benjamini–Hochberg false discovery rate procedure, applied by the type of outcome (spatial–cognitive, motor–cognitive, motor, and cognitive) for multiple corrections. The false discovery rate, alpha, was set as *p* < 0.05. The Arsenal tableby function via R Studio (R version 4.3.3) was utilized to compare outcome variables between the two onset groups. Effect sizes are calculated using Cohen’s d.

#### Exploratory Stratified Analysis Based on Level of Functional Impairment

Because onset-based differences in spatial–cognitive performance may depend on the level of impairment, the sample was further analyzed after stratification by three different categories reflecting level of functional impairment: symptom lateralization status, level of cognitive impairment, and level of freezing of gait (FOG). Symptom lateralization status was defined by the Hoehn and Yahr (H&Y) scale and was scored within the Unified Parkinson’s Disease Rating Scale (UPDRS): 1 and 1.5 = unilateral symptoms, 2 = bilateral symptoms, and 2.5 = bilateral symptoms with postural instability. Level of cognitive impairment was defined by Montreal Cognitive Assessment total score: >23 = normal cognition, 18~25 = mild cognitive impairment, and <18 = moderate to severe dementia [55]. Level of freezing of gait was defined by UPDRS item 3.11 and Freezing of Gait Questionnaire question 3: 0 (UPDRS) and 0 (FOGQ) = non-freezer, >0 (UPDRS) and >0 (FOGQ) = freezer, and >0 (UPDRS) and >2 (FOGQ) = frequent freezer. In question 3 of FOGQ, 0 indicates no freezing at all, 1 indicates freezing occasionally, and 2 indicates often. In item 3.11 of UPDRS, 0 indicates no freezing, and 1 indicates slight freezing (see Appendix B for scoring criteria) [61]. To analyze the effect of side of onset on spatial–cognitive and motor–cognitive outcomes for each stratified subgroup, each sample was tested using the Mann–Whitney U test. *p*-values for subgroup analyses were not corrected due to the exploratory nature and small sample size; thus, cautious interpretation is needed. Effect sizes are calculated using the rank-biserial correction r.

## 3. Results

Clinical and demographic characteristics are presented in Table 1. About half (50.46%) of the participants had ROPD. ROPD reported a significantly higher level of freezing from the Freezing of Gait Questionnaire (R = 1.37 ± 1.3, L = 0.91 ± 1.3, *p* = 0.008). The mean level of education was higher than a master’s degree or equivalent for both groups (ROPD = 15.94 ± 2.6; LOPD = 16.57 ± 2.2). ROPD had a slightly longer duration of PD (8.06 ± 6.1) than LOPD (6.81 ± 4.9). Both groups had an H&Y stage of 2 and moderate functioning according to the composite physical function score (ROPD = 19.21 ± 4.9; LOPD = 19.55 ± 5.5). In both groups, there were more males than females, more white people than other racial groups, more right-handed people, and more people who do not use an assistive device to walk (Table 1).

Across the entire sample, there were no significant differences in any spatial–cognitive, motor–cognitive, motor, or cognitive outcomes between LOPD and ROPD (Table 2).

**Figure 1:**
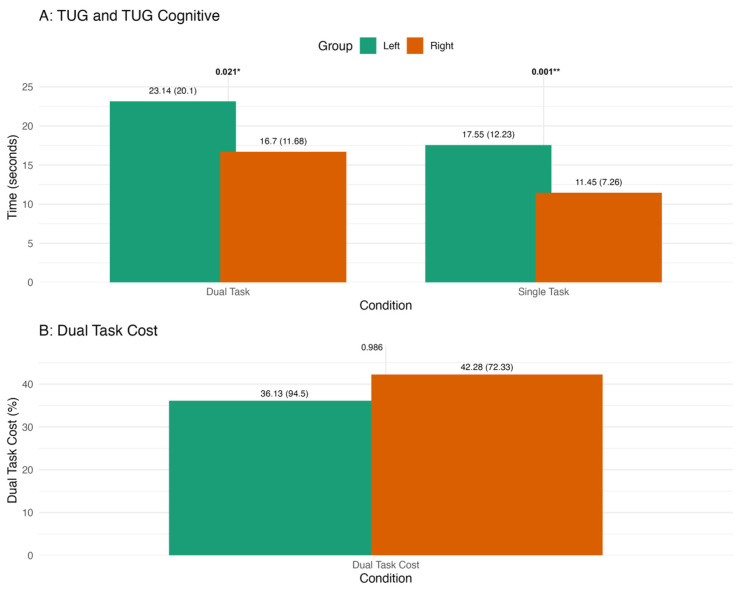
Bars plotting median (IQR) values compared between left-onset (green) and right-onset (orange) PD with both bilateral symptoms and postural instability in time taken to perform single-task and dual-task TUG (**A**) and percentage value of dual-task cost (**B**). *p*-values on the top row indicate significant left-onset vs. right-onset difference for both single- and dual-task TUG, but the bottom row shows no significant difference for dual-task cost. * *p* < 0.05. ** *p* < 0.01.

In people with only unilateral and only bilateral PD symptoms, we found no significant difference between LOPD vs. ROPD in any outcome (Table 3a,b). However, in people with both bilateral symptoms and postural instability, LOPD performed worse in both single-task (*p* = 0.001) and dual-task (*p* = 0.021) TUG, but there was no significant difference in the dual-task cost (Table 3c) (Figure 1).

There was no significant difference in any outcome between onset groups in people with normal cognition or with MCI. In people with MoCA scores of 18 and below, LOPD performed worse in single-task TUG (*p* = 0.036), but there were no significant differences in dual-task TUG or DTC value (Table 4). LOPD also performed worse in FSST, as four out of five participants were unable to attempt the task due to physical difficulties, and the remaining ROPD had a longer completion time.

As ROPD scored significantly higher in FOGQ #3 than LOPD, we wanted to isolate people experiencing freezing of gait by freezers, frequent freezers, and non-freezers (see Section 2). There was no significant difference in spatial–cognitive and motor–cognitive performance between LOPD and ROPD among non-freezers. In freezers, LOPD performed significantly worse in single-task TUG, but there was no significant difference in TUG dual-task or DTC (Table 5a). In frequent freezers, though LOPD performed worse in single- and dual-task TUG than ROPD, the differences were not significant. However, LOPD had a significantly larger TUG-DTC (Table 5b).

## 4. Discussion

Consistent with the literature, our results also show that while there was no significant effect of side of onset on spatial–cognitive and motor–cognitive outcomes generally [34], LOPD tends to perform worse in TUG tasks among subgroups of those with more severe symptoms [37]. This applied to both motor and cognitive symptoms, as significant LOPD vs. ROPD differences emerged in both TUG-Simple and TUG-Cognitive for groups with bilateral symptoms and postural instability; in TUG-Simple for groups with MoCA scores lower than 18 and groups that freeze; and in TUG-DTC for groups that freeze more frequently. One possible interpretation is that spatial cognition is affected by the side of PD onset, but while the effect does not show in spatial tasks directly, it rather shows in motor–cognitive dual tasks that use spatial cognition. Future studies should use mediation analysis to investigate the possibility of spatial cognition serving as a mediator of the effect of the side of onset on functional performance in people with PD.

While TUG-Cognitive serves as a raw performance value during a motor–cognitive task, TUG-DTC demonstrates dual-task interference, which indicates slower or decreased performance when trying to complete two tasks simultaneously [62]. While a significantly worse TUG-Cognitive performance by LOPD was present for those having bilateral PD symptoms with postural instability, it is difficult to interpret that the dual-tasking ability was affected for the subgroup, as there was no significant DTC value. Frequent freezing did reveal an effect of the side of onset on TUG-DTC but not on other values, which could suggest that there was a difference in dual-task interference but not in the raw performance in each task. Both TUG-Simple and TUG-Cognitive are known to detect fall risk [57,63], functional mobility [60,64], and cognitive decline [28,65,66]; no significant difference in sensitivity to cognitive decline was found between the two tests [67]. As such, the results suggest that freezing, bilateral PD symptoms with postural instability, and moderate cognitive impairment affect simple mobility, potentially extending effects to fall risk and cognitive decline.

However, the results for these subgroup comparisons must be interpreted with much caution due to the limited sample size for each. Sample sizes for the stratified subgroups—especially both LOPD and ROPD groups of frequent freezers (LOPD, N = 9; ROPD, N = 14), those with MoCA scores lower than 18 (LOPD, N = 5; ROPD, N = 4), and the LOPD group of freezers (N = 14)—are very small, presenting a major limitation to this study. We emphasize that the design and results of this study are highly exploratory, and thus the findings should be interpreted very cautiously.

Our finding of worse TUG performance by freezers also supports spatial impairment’s association with motor functioning and freezing of gait shown in the previous literature [21,22,23]. Different TUG-related outcomes showed a significant effect of the side of PD onset in groups of different freezing severities. Validation via a larger sample size is needed to confirm the results presented here. As such, this inquiry of which and whether specific PD characteristics reveal functional lateralization can become an area for future research that could further provide insight into PD disease progression, such as patterns of how and when different functionalities decline.

Notably, while the effect of the side of onset was consistently present in TUG tasks, it was not present in any other outcomes. TUG-Simple, compared to the other motor-based tasks, involves bigger and a greater variety of movement, such as sitting up and down, walking a longer 3 m distance, and turning, and TUG-Cognitive explicitly adds dual-tasking burden with a cognitive task on top of the motor task. ROPD has traditionally been found to have worse motor symptom prognosis [68] and worse impact of medication-related motor fluctuation in daily life compared to LOPD [35]. Yet, these studies did not examine the impact of motor–cognitive dual-tasking nor the type of movement affected. Future research on the side of onset differences in specific motor symptoms may provide further insight into explaining these results.

It is also important to note that, overall, different categories of subgroups showed significant side of onset differences in different types of TUG tasks. These results do not present rigid conclusions due to the exploratory nature of the analyses, including no multiple subgroup comparisons and the small subgroup sample sizes, but the purpose of presenting these results is to inspire and guide future research on these stratified approaches to the effect of the side of PD onset.

Our results are inconsistent with Seichepine et al. (2016), who found worse spatial–cognitive performance by LOPD than ROPD in the clock drawing test [42]. On the other hand, they were consistent with Yoon et al. (2016) and Adwani et al. (2016), who found no effect of side of onset in samples of less severe disability, such as H&Y score lower than 2.5 and lower than 2, respectively [43,45]. However, our study did not find any LOPD vs. ROPD difference in similar visuospatial–cognitive tasks, such as MoCA clock and cube drawing tasks, in people with higher H&Y stages or higher levels of other categories of impairment. Therefore, the effect of the side of onset may be nondetectable with these shape-based visuospatial–cognitive tasks or absent in abilities required by those tasks.

Similarly, Karádi et al. (2015) identified a significant effect of the side of onset in visuoconstructional tasks only in people with longer PD duration, which further emphasizes the level of deterioration as a factor in revealing side of onset differences [44]. In our study, post hoc analysis comparing LOPD vs. ROPD on spatial–cognitive outcomes based on subgroups of shorter (<6) and longer (>5) PD duration did not reveal significant differences. As PD is a highly heterogeneous disease, it may be that PD duration may not be reflective of the actual severity of symptoms and functional disability, and it is this severity that influences symptom lateralization in spatial cognition.

A meta-analysis with around 1000 LOPD and 1000 ROPD suggests no significant effect of side of onset on PD symptom severity, which is in alignment with our result [40]. However, our study expands beyond the scope of this meta-analysis to compare within similar symptom severity; thus, it is a novel approach. Due to our study’s major limitation of subgroups’ small sample sizes, furthering this approach with larger samples is essential for meaningful interpretation. Again, with more power, examining the effect of the side of onset across different levels of symptomatology of PD may provide insight into the underlying mechanism of cognitive decline in PD.

PD is suggested to typically have a prodromal stage during which classic motor symptoms sufficient for PD diagnosis are absent, but nonmotor symptoms, including cognitive, psychosocial, and sleep issues, arise. This stage can last from five to 20 years [69]. Therefore, even if a lateralized pattern of cognitive decline in PD was apparent in the earlier prodromal stage, it could have been masked, advancing onto bilateral affection, by the time of diagnosis. Investigating the effect of the side of onset on cognitive decline at the prodromal stage may provide further insight into how the disease affects the brain and its function.

This study provides a large dataset of clinical and demographic outcomes of people with PD and presents these findings in the context of functional specialization, which has received little attention lately in research on neurodegenerative disease. Future studies that integrate functional specialization in PD with imaging-based neural mapping may provide further insight into side of onset’s relationship with spatial function, as well as other domains of cognitive function. Further, the study provides unique insights into the effect of the side of onset on outcomes related to more severe symptomatology, e.g., cognitive impairment, freezing, and stage of disease.

## Figures and Tables

**Table 1:** Demographic characteristics in left- vs. right-onset PD.

	Left	Right	*p*-Value
N (%)	107 (49.54)	109 (50.46)	
Age (Years)	69.80 ± 8.5	69.60 ± 7.8	0.858
Education Level (Years)	16.57 ± 2.2	15.94 ± 2.6	0.060
BMI	26.61 ± 5.6	26.82 ± 5.0	0.774
Year with PD	6.81 ± 4.9	8.06 ± 6.1	0.103
Number of Comorbidities	3.48 ± 1.7	3.68 ± 2.2	0.464
Composite Physical Function (/24)	19.55 ± 5.5	19.21 ± 4.9	0.632
MDS-UPDRS I	10.38 ± 7.3	10.73 ± 8.0	0.742
MDS-UPDRS II	12.54 ± 8.9	14.53 ± 8.6	0.102
MDS-UPDRS III	33.75 ± 15.7	33.15 ± 13.0	0.760
MDS-UPDRS IV	4.48 ± 3.6	5.13 ± 3.8	0.200
Freezing of Gait Questionnaire #3 (/4) *	0.91 ± 1.3	1.37 ± 1.3	0.008
Hoehn & Yahr, Median (IQR) *^	2 (1.0)	2 (0.5)	0.166
**Sex**			0.062
Female (%)	44 (41.5)	32 (29.4)	
Male (%)	62 (58.5)	77 (70.6)	
**Ethnicity**			0.290
White	78 (73.6%)	80 (74.1%)	
Black	19 (17.9%)	19 (17.6%)	
Asian	3 (2.8%)	0 (0.0%)	
Hispanic/Latino	0 (0.0%)	3 (2.8%)	
Native American	0 (0.0%)	1 (0.9%)	
Multiracial	4 (3.8%)	4 (3.7%)	
Other	2 (1.9%)	1 (0.9%)	
**Handedness**			0.062
Left	17 (16.2)	8 (7.8)	
Right	88 (83.8)	95 (92.2)	
**Use of Assistive Device,** ***n*** **(%)**			0.291
No	60 (57.7)	72 (66.7)	
Sometimes	18 (17.3)	18 (16.7)	
Yes	26 (21.1)	18 (16.7)	

Mean ± SD for continuous variables, and *n* (%) for categorical variables. ^ Hoehn and Yahr score presented by median (IQR) and tested by the Mann–Whitney U test (W = 4878). *p*-values calculated with independent *t*-tests for continuous variables and the chi-square test for categorical variables. * *p* < 0.05; see Appendix B for freezing of gait scoring criteria.

**Table 2:** Spatial, motor, motor–cognitive, and cognitive performance in the left versus right side of PD onset.

	Left	Right	*p*-Value	d
Body Position Spatial Task (BPST) Correct Trials	4.48 (1.4)	4.27 (1.6)	0.793	0.131
BPST Span	3.78 (0.9)	3.77 (1.1)	1.000	0.017
Corsi Blocks Correct Trials	5.82 (1.9)	5.97 (2.3)	0.830	0.073
Corsi Blocks Span	4.32 (1.1)	4.52 (1.5)	0.793	0.146
MoCA Visuospatial Score	3.91 (1.3)	3.79 (1.1)	0.817	0.100
MoCA Clock Drawing Total Score	2.48 (0.81)	2.48 (0.75)	1.000	0
MoCA Cube Drawing Score	0.58 (0.50)	0.49 (0.50)	0.793	0.170
MoCA Total Score	25.68 (3.7)	25.47 (3.5)	0.673	0.058
Four Square Step Test (FSST) (s)	11.55 (4.4)	12.86 (7.1)	0.672	0.219
TUG-Simple (s)	13.78 (16.6)	11.80 (7.2)	0.262	0.155
TUG-Cognitive (s)	18.73 (18.6)	17.97 (14.3)	0.846	0.046
TUG-DTC (%)	49.97 (54.0)	48.46 (57.9)	0.846	0.027
Trail A (s)	42.09 (21.1)	40.85 (21.7)	0.846	0.058
Trail B (s)	111.46 (80.5)	118.31 (80.7)	0.846	0.085
Trail B − Trail A (s)	68.03 (64.5)	74.81 (62.5)	0.846	0.107

Values are presented as the mean (SD). *p*-values calculated with an independent *t*-test. *p*-values adjusted using the Benjamini–Hochberg procedure for multiple comparison corrections. *p* < 0.05. Effect size calculated with Cohen’s d.

**Table 3:** (**a**) Spatial, motor, motor–cognitive, and cognitive performance in the left versus right side of PD onset in people with unilateral symptoms (H&Y < 2). (**b**) Spatial, motor, motor–cognitive, and cognitive performance in the left versus right side of PD onset in people with bilateral symptoms (H&Y > 1.5). (**c**) Spatial, motor, motor–cognitive, and cognitive performance in the left versus right side of PD onset in people with bilateral symptoms and postural instability (H&Y > 2).

(**a**)					
	**Left (*****n*** **= 28)**	**Right (*****n*** **= 21)**	**W**	***p*** **-Value**	**r**
BPST Correct Trials	4.75 (1.43)	4.33 (1.77)	331.5	0.442	0.128
BPST Span	3.82 (0.72)	3.76 (1.09)	289.5	0.932	−0.015
Corsi Blocks Correct Trials	6.57 (1.71)	5.86 (2.56)	348.0	0.274	0.184
Corsi Blocks Span	4.79 (1.23)	4.43 (1.5)	329.5	0.466	0.121
MoCA Visuospatial Score	4.43 (1.14)	4.05 (1.02)	373.0	0.073	0.269
MoCA Clock Drawing Total Score	2.67 (0.78)	2.67 (0.48)	315.0	0.394	0.111
MoCA Cube Drawing Score	0.74 (0.45)	0.65 (0.50)	318.0	0.379	0.122
MoCA Total Score	27.64 (2.33)	26.62 (2.87)	358.5	0.190	0.219
FSST (s)	9.35 (1.77)	10.02 (3)	273.5	0.686	−0.070
TUG-Simple (s)	8.54 (1.88)	9.01 (2.32)	272.0	0.667	−0.075
TUG-Cognitive (s)	12.53 (4.62)	12.12 (4.03)	291.0	0.828	0.039
TUG-DTC	51.19 (65.06)	32.1 (21.19)	312.0	0.514	0.114
Trail A (s)	29.4 (11.02)	28.91 (14.65)	324.0	0.551	0.102
Trail B (s)	67.95 (30.19)	77.76 (60.94)	300.0	0.912	0.020
Trail B − Trail A (s)	38.55 (26.27)	48.85 (48.4)	287.0	0.897	−0.024
(**b**)					
	**Left (*****n*** **= 77)**	**Right (*****n*** **= 83)**	**W**	***p*** **-Value**	**r**
BPST Correct Trials	4.33 (1.41)	4.29 (1.62)	3272.0	0.579	0.050
BPST Span	3.74 (0.88)	3.77 (1.07)	3200.0	0.76	0.027
Corsi Blocks Correct Trials	5.57 (1.90)	6.05 (2.26)	2682.0	0.128	−0.138
Corsi Blocks Span	4.16 (1.07)	4.59 (1.59)	2622.0	0.075	−0.158
MoCA Visuospatial Score	3.71 (1.28)	3.76 (1.16)	3145.5	0.969	−0.004
MoCA Clock Drawing Total Score	2.43 (0.80)	2.43 (0.81)	3104.0	0.956	−0.005
MoCA Cube Drawing Score	0.51 (0.50)	0.48 (0.50)	3196.5	0.756	0.025
MoCA Total Score	24.99 (3.94)	25.51 (3.28)	2994.0	0.574	−0.052
FSST (s)	12.42 (4.84)	13.65 (7.86)	2694.0	0.504	−0.063
TUG-Simple (s)	15.8 (19.21)	12.22 (7.7)	3465.5	0.132	0.140
TUG-Cognitive (s)	21.17 (21.41)	18.73 (15.4)	3245.5	0.24	0.110
TUG-DTC	49.4 (49.19)	50.6 (63.2)	2993.0	0.8	0.024
Trail A (s)	47.03 (22.08)	42.94 (22.23)	3643.5	0.126	0.140
Trail B (s)	128.59 (87.75)	124.76 (79.82)	3199.5	0.877	0.014
Trail B − Trail A (s)	79.64 (71.36)	79.05 (62.84)	3048.0	0.716	−0.034
(**c**)					
	**Left (*****n*** **= 33)**	**Right (*****n*** **= 45)**	**W**	***p*** **-Value**	**r**
BPST Correct Trials	4.09 (1.33)	3.93 (1.34)	762.5	0.529	0.083
BPST Span	3.59 (0.91)	3.52 (0.88)	737.5	0.712	−0.199
Corsi Blocks Correct Trials	5.06 (1.48)	5.82 (2)	569.5	0.073	−0.233
Corsi Blocks Span	3.88 (0.82)	4.33 (1.4)	595.0	0.116	−0.199
MoCA Visuospatial Score	3.33 (1.47)	3.41 (1.21)	715.5	0.916	−0.014
MoCA Clock Drawing Total Score	2.09 (2.30)	0.95 (0.85)	641.0	0.348	−0.117
MoCA Cube Drawing Score	0.52 (0.36)	0.51 (0.49)	836.0	0.189	0.152
MoCA Total Score	23.76 (4.71)	24.95 (3.31)	643.0	0.394	−0.144
FSST (s)	15.8 (6.01)	15.86 (9.47)	661.5	0.331	0.140
TUG-Simple (s) **	24.29 (27.44)	14.49 (9.8)	963.5	0.001	0.434
TUG-Cognitive (s) *	32.49 (30.03)	23.77 (19.52)	832.0	0.021	0.321
TUG-DTC	61.17 (64.94)	61.56 (76.81)	632.0	0.986	0.003
Trail A (s)	55.59 (22.47)	50.42 (24.56)	883.0	0.157	0.189
Trail B (s)	163.13 (90.98)	157.01 (84.92)	742.0	0.824	0.031
Trail B − Trail A (s)	102.94 (74.47)	101.5 (68.59)	706.0	0.890	−0.019

Mean (SD). *p*-values calculated with the Mann–Whitney U test. * *p* < 0.05. ** *p* < 0.01. Effect size calculated with the rank-biserial correlation coefficient r.

**Table 4:** Spatial, motor, motor–cognitive, and cognitive performance in the left versus right side of PD onset in people with MoCA scores < 18.

	Left (*n* = 5)	Right (*n* = 4)	W	*p*-Value	r
BPST Correct Trials	2.75 (1.0)	2.75 (1.0)	8	1.000	0
BPST Span	2.75 (1.0)	2.75 (1.0)	8	1.000	0
Corsi Blocks Correct Trials	4.40 (2.2)	4.25 (0.5)	12.5	0.602	0.250
Corsi Blocks Span	3.60 (1.1)	3.50 (0.6)	11	0.896	0.100
MoCA Visuospatial Score	1.20 (1.3)	2.00 (0.8)	6	0.377	−0.400
MoCA Clock Drawing Total Score	1.00 (1.00)	2.00 (0.82)	4.5	0.197	−0.550
MoCA Cube Drawing Score	0.20 (0.45)	0.00 (0.00)	12	0.502	0.200
MoCA Total Score	15.20 (1.8)	15.25 (2.2)	8.5	0.770	−0.150
FSST (s)	31.97 (NA ^^^)	17.18 (6.7)	4	0.400	1.000
TUG-Simple (s) *	32.98 (9.9)	12.61 (1.5)	15	0.036	1.000
TUG-Cognitive (s)	23.14 (20.1)	18.10 (4.2)	12	0.057	1.000
TUG-DTC	36.13 (94.5)	42.28 (72.33)	6	1.000	0.000
Trail A (s)	54.33 (30.55)	44.9 (26.57)	12	0.687	0.200
Trail B (s)	133.63 (172.56)	134.18 (119.46)	8.5	1.000	0.062
Trail B − Trail A (s)	81.33 (120.74)	84.93 (104.54)	8	1.000	0.000

Mean (SD). *p*-values calculated with the Mann–Whitney U test. * *p* < 0.05. Effect size calculated with the rank-biserial correlation coefficient r. ^ No SD value because 4 out of 5 participants were unable to attempt task due to physical difficulties (i.e., fatigue, use of walker, lack of confidence).

**Table 5:** (**a**) Spatial, motor, motor–cognitive, and cognitive outcomes in the left vs. right side of onset in all freezers (FOGQ > 0, UPDRS 3.11 > 0). (**b**) Spatial, motor, motor–cognitive, and cognitive outcomes in the left vs. right side of onset in frequent freezers (FOGQ #3 > 2, UPDRS 3.11 > 0).

(**a**)					
	**Left (*****n*** **= 14)**	**Right (*****n*** **= 34)**	**W**	***p*** **-Value**	**r**
BPST Correct Trials	4.36 (1.34)	4.36 (1.64)	244.0	0.766	0.056
BPST Span	3.64 (0.84)	3.67 (1.02)	236.0	0.912	0.022
Corsi Blocks Trial	5.00 (1.3)	5.47 (2.3)	199.5	0.378	−0.162
Corsi Blocks Span	3.79 (0.7)	4.09 (1.33)	194.0	0.298	−0.185
MoCA Visuospatial Score	3.64 (1.08)	3.67 (0.92)	232.5	0.98	0.006
MoCA Clock Drawing Total Score	2.21 (0.89)	2.52 (0.62)	192.5	0.322	−0.167
MoCA Cube Drawing Score	0.57 (0.51)	0.42 (0.50)	265.0	0.367	0.147
MoCA Total Score	25.00 (2.75)	25.24 (2.96)	206.0	0.564	−0.108
FSST (s)	15.54 (6.25)	15.30 (11.1)	228.5	0.343	0.190
TUG-Simple (s) *	28.39 (41.88)	14.48 (11.07)	279.0	0.048	0.385
TUG-Cognitive (s)	35.44 (42.5)	21.96 (16.89)	257.0	0.056	0.382
TUG-Dual-Task Cost	85.73 (76.41)	52.67 (46.20)	218.0	0.394	0.172
Trail A (s)	52.50 (25.78)	42.94 (18.09)	306.0	0.126	0.286
Trail B (s)	148.32 (82.25)	131.97 (76)	268.0	0.503	0.126
Trail B − Trail A (s)	97.16 (66.88)	88.46 (61.41)	253.0	0.742	0.063
(**b**)					
	**Left (*****n*** **= 9)**	**Right (*****n*** **= 14)**	**W**	***p*** **-Value**	**r**
BPST Correct Trials	4.11 (1.54)	4.31 (1.93)	58	0.802	−0.009
BPST Span	3.56 (1.01)	3.54 (1.13)	60	0.971	0.026
Corsi Blocks Trial	5.11 (1.27)	6.36 (2.13)	45.0	0.255	−0.286
Corsi Blocks Span	3.78 (0.67)	4.50 (0.94)	36.5	0.069	−0.421
MoCA Visuospatial Score	3.78 (0.97)	3.69 (1.11)	60.0	0.944	0.026
MoCA Clock Drawing Total Score	2.33 (0.87)	2.38 (0.77)	57.5	0.970	−0.017
MoCA Cube Drawing Score	0.67 (0.50)	0.46 (0.52)	70.5	0.374	0.205
MoCA Total Score	25.11 (2.93)	25.54 (2.76)	52	0.731	−0.111
FSST (s)	15.84 (6.41)	14.55 (8.03)	52.0	0.432	0.238
TUG-Simple (s)	36.23 (52.73)	16.95 (15.46)	68.0	0.135	0.417
TUG-Cognitive (s)	46.29 (54.07)	22.47 (16.56)	62.0	0.1	0.476
TUG-Dual-Task Cost *	120.62 (70.34)	42.64 (28.92)	72.0	0.01	0.714
Trail A (s)	50.19 (19.07)	39.19 (16)	92.0	0.072	0.460
Trail B (s)	139.57 (87.61)	141.76 (89.4)	60.5	0.9	−0.040
Trail B − Trail A (s)	91.45 (79.28)	102.57 (75.68)	55.0	0.643	−0.127

Mean (SD). *p*-values calculated with the Mann–Whitney U test. * *p* < 0.05. Effect size calculated with the rank-biserial correlation coefficient r.

## Data Availability

The original contributions presented in this study are included in the article. Further inquiries can be directed to the corresponding author.

## Abbreviations

The following abbreviations are used in this manuscript:

UPDRS	Unified Parkinson’s Disease Rating Scale
FOGQ	Freezing of Gait Questionnaire
PD	Parkinson’s Disease
TUG	Timed Up and Go

## Appendix A

### Appendix A.1. Spatial Cognition Task Description

The body position spatial task (BPST) tests spatial memory and navigation using the whole body. The examiner verbally and visually demonstrates a pattern of side, forward, and/or turning steps, which the examinee repeats after watching once [6].

The Reverse Corsi Blocks test assesses visuospatial memory, as the examiner taps a series of blocks in a certain order, and the participant then repeats this action in the reverse order [54].

While the MoCA as a whole assesses global cognitive impairment of the individual, its first three items assess visuospatial function, focusing on attention and construction [55]. Its items include a shorter, 10-item version of the trails B test, copying a box drawing, and drawing a clock with a given time.

### Appendix A.2. Motor Cognition Task Description

The Four Square Step Test (FSST) assesses motor navigation and mobility, as the participant is required to step onto each tile of a window-shaped square on the ground in one direction and then its reverse, as quickly and safely as possible [58].

The trails test examines executive function, working memory, and visuomotor speed, as participants draw a line connecting widespread bubbles of different numbers, in ascending numerical order (part A). In Part B, bubbles contain either a number or a letter, and participants must connect the bubbles in alternating, ascending order [59].

The Timed Up and Go (TUG) test is a measure of motor cognition that has two parts: a single-task TUG test that tests mobility [60] and a dual-task TUG test that tests the ability to dual-task between motor and cognitive activities [57]. In the single-task TUG, the participant stands up from a chair, walks 3 m, turns around, walks back, and sits down. The same motor task is done while counting backwards by 3s from 100 for the dual-task TUG. The dual-task cost (DTC) of TUG is a valid and reliable measure of functional mobility in PD and indicates the percent change in time to complete the dual task versus the single task [56]. See the equation below. DTC incorporates both motor and cognitive ability and involves the lower body, as well as demonstrating detection of fall risk [28,29,69].

DTC = (TUG-Cognitive − TUG-Single)/TUG-Single.

## Appendix B

### Appendix B.1. UPDRS 3.11 Scoring Criteria

0: Normal: No freezing.
1: Slight: Freezes on starting, turning, or walking through the doorway with a single halt during any of these events but then continues smoothly without freezing during straight walking.
2: Mild: Freezes on starting, turning, or walking through the doorway with more than one halt during any of these activities but continues smoothly without freezing during straight walking.
3: Moderate: Freezes once during straight walking.
4: Severe: Freezes multiple times during straight walking.

### Appendix B.2. FOGQ #3 Scoring Criteria

**Question**: “Do you feel that your feet get glued to the floor while walking, making a turn or when trying to initiate walking?”
0: Never.
1: Rarely (e.g., 1–2 times a month).
2: Often (e.g., about once a day).
3: Very often (e.g., more than once a day).
4: Always (e.g., whenever walking).
